# Cover Cropping Alters the Diet of Arthropods in a Banana Plantation: A Metabarcoding Approach

**DOI:** 10.1371/journal.pone.0093740

**Published:** 2014-04-02

**Authors:** Gregory Mollot, Pierre-François Duyck, Pierre Lefeuvre, Françoise Lescourret, Jean-François Martin, Sylvain Piry, Elsa Canard, Philippe Tixier

**Affiliations:** 1 CIRAD, UR 26 Systèmes de culture à base de bananiers, plantains et ananas, PRAM, Le Lamentin, Martinique, France; 2 INRA, UR-1115 Plantes et Systèmes de culture Horticoles, Avignon, France; 3 CIRAD, UMR PVBMT, CIRAD/Université de La Réunion, Pôle de Protection des Plantes, Saint-Pierre, La Réunion, France; 4 Montpellier-SupAgro, UMR CBGP, Montferrier-sur-Lez, France; 5 INRA, UMR1062 CBGP, Montferrier-sur-Lez, France; 6 CNRS-IRD, UMR 2724 MIVEGEC, Montpellier, France; 7 CIRAD – CATIE, Departamento de Agricultura y Agroforesteria, CATIE, Turrialba, Costa Rica; Pennsylvania State University, United States of America

## Abstract

Plant diversification using cover crops may promote natural regulation of agricultural pests by supporting alternative prey that enable the increase of arthropod predator densities. However, the changes in the specific composition of predator diet induced by cover cropping are poorly understood. Here, we hypothesized that the cover crop can significantly alter the diet of predators in agroecosystems. The cover crop *Brachiaria decumbens* is increasingly used in banana plantations to control weeds and improve physical soil properties. In this paper, we used a DNA metabarcoding approach for the molecular analysis of the gut contents of predators (based on mini-COI) to identify 1) the DNA sequences of their prey, 2) the predators of *Cosmopolites sordidus* (a major pest of banana crops), and 3) the difference in the specific composition of predator diets between a bare soil plot (BSP) and a cover cropped plot (CCP) in a banana plantation. The earwig *Euborellia caraibea*, the carpenter ant *Camponotus sexguttatus*, and the fire ant *Solenopsis geminata* were found to contain *C. sordidus* DNA at frequencies ranging from 1 to 7%. While the frequencies of predators positive for *C. sordidus* DNA did not significantly differ between BSP and CCP, the frequency at which *E. caraibea* was positive for Diptera was 26% in BSP and 80% in CCP; the frequency at which *C. sexguttatus* was positive for *Jalysus spinosus* was 14% in BSP and 0% in CCP; and the frequency at which *S. geminata* was positive for *Polytus mellerborgi* was 21% in BSP and 3% in CCP. *E. caraibea*, *C. sexguttatus* and *S. geminata* were identified as possible biological agents for the regulation of *C. sordidus*. The detection of the diet changes of these predators when a cover crop is planted indicates the possible negative effects on pest regulation if predators switch to forage on alternative prey.

## Introduction

Agriculture faces the challenges of providing more food and energy while adapting to climate change and mitigating environmental impacts. One of the most promising approaches to meet these challenges is the design of new agroecosystems based on the management of ecological processes rather than on application of fertilizers and pesticides [1]. For instance, the regulation of crop pests through top-down and bottom-up effects remains a potential alternative to reduce the ecological imprints of agroecosystems while maintaining production [2]. This regulation could rely on the management of primary resources, such as the addition of cover crops [3]. The underlying hypothesis is that cover crops enable the development of alternative preys leading to higher densities and diversities of generalist arthropod predators. The larger the densities of predators, the higher the consumption of herbivore pests - provided that the pest remains a favourite prey [4], [5]. It follows that designing environmentally friendly cropping systems requires a clear understanding of food web functions.

In spite of the substantial research conducted in the last decade, food web ecology suffers from a lack of efficient and comprehensive methods to measure trophic links *in natura* with accuracy. To date, trophic links are often inferred by abundance measurements of predators and prey [6]–[10], stable isotope analyses [11]–[17], and protein-based approaches [18], [19]. Although recent molecular approaches, like multiplex-PCR, have enabled identification of specific prey in the gut contents and faeces of a wide range of predators [20], these methods are not suited for the detection of unexpected prey [21]. Recently, Next Generation Sequencing (NGS) technology has been used to examine the specific composition of the diet of a given predator or herbivore and to describe two-level food webs [for a review, see 22]. The development of DNA metabarcoding now enables researchers to measure trophic links without *a priori* knowledge of the consumed species and to determine the diet of each individual [22]. The metabarcoding approach is based on the pyrosequencing of a DNA barcode (amplified with universal primers) that can discriminate and identify species in a DNA mixture. This method could be used to analyse gut contents of arthropods, because it has the potential to identify the complex diet of generalist predators.

Plant diversification has strong bottom-up effects on multitrophic interaction networks, especially on lower trophic levels [23], but it remains unclear whether the addition of a cover crop in agroecosystems actually leads to enhanced pest control by predators [24]. In banana agroecosystems in Martinique, the cover crop *Brachiaria decumbens* Stapf, a tropical C4 grass, is increasingly used to control weeds and to improve physical soil properties. In these agroecosystems, Mollot *et al.* [25] showed that *Solenopsis geminata* (F.), a generalist predator feeding on the banana weevil *Cosmopolites sordidus* (Germar), the major banana insect pest, was more abundant in cover cropped plots (CCP) than in bare soil plots (BSP). Along with this increase in densities, *S. geminata* exhibited a change in isotopic signature, indicating that it fed on the C4 pathway provided by the new resource. In the same study, the monitoring of eggs of *C. sordidus* artificially deposited in plots showed that predation on eggs was always higher in CCP than in BSP. However, the specific changes in the diets of predators affected by the cover crop are unclear. Identifying the prey consumed and determining the rate at which they are consumed by the major generalist predators would help us understand the effects of the cover crop on predator diets and thus on the regulation of *C. sordidus*.

Using a metabarcoding approach based on the COI barcode, we assessed the diet of eight ground-dwelling predators commonly found in banana plantations in Martinique: wolf spiders from the Lycosidae family, the earwig *Euborellia caraibea* Hebard, the carpenter ant *Camponotus sexguttatus* (F.), the trap jaw ant *Odontomachus baurii* Emery, the fire ant *S. geminata*, the little fire ant *Wasmannia auropunctata* (Roger), rove beetles from the Staphilinidae family, and centipedes from the Scolopendridae family. We amplified a shortened fragment of COI (mitochondrial cytochrome *c* oxidase I) from the gut contents of predators to identify (1) DNA sequences of their prey, (2) predators of *C. sordidus*, and (3) the difference in predator diet between CCP and BSP. Based on these results, we make suggestions about how to use and manage a *B. decumbens* cover crop to control the populations of *C. sordidus* in banana plantations. Finally, we discuss the technical implications of the use of the metabarcoding approach to assess the diet of ground-dwelling predators.

## Materials and Methods

### Ethics Statement

All of the authors declare that the experiments performed in the present study comply with the current laws of France. No specific permits were required for the described field study, which involved sampling of invertebrates and plant species. No specific permits were required to perform the described study in this location, which is an experimental farm owned by CIRAD. All of the authors confirm that the location is not privately owned or protected in any way and that the field studies did not involve endangered or protected species.

### Study Sites

Sampling was conducted in Martinique (French West Indies) between January and June 2011. Samples were collected from an experimental farm in Rivière Lézarde (14°39′45.04″N; 60°59′59.08″W) in two adjacent plots: a bare soil plot (BSP) of 300 m^2^ and a *B. decumbens* cover cropped plot (CCP) of 368 m^2^. Both plots were in the sixth year of banana production without insecticide application; plants were unsynchronized and harvested throughout the year.

### Sampling

The first step of the procedure was the construction of a reference bank of DNA sequences that included every possible arthropod prey taxa from the studied sites. To construct this bank of sequences, we designed a sampling scheme to capture most of the arthropod diversity in banana agroecosystems. We collected two to four samples (one sample corresponds to one individual of a given taxon) belonging to each of 15 taxa commonly found in banana fields (taxa and trapping methods are listed in **Table S1**). Soil-surface arthropod samples were collected with dry pitfall traps and pseudostem traps (one-half of a section of fresh banana pseudostem, 50 cm long), whereas flying arthropod samples (*Gryllus*, Cicadellidae, and Pentatomidae) were collected by 15-s sessions with a suction sampler (D-vac, Rincon-Vitova Insectaries, Inc., Ventura, California, USA). We also directly collected samples with clean forceps to obtain arthropods that were not trapped by pitfall traps, pseudostem traps, or vacuum sampling.

The samples for the diet analyses were obtained by collecting individual samples from the most common ground-dwelling predators (*n* = 572) (**Table S2**). The recovery of DNA from the gut contents of predators was optimized by placing samples in a portable cooler (4°C) in the field so as to decrease enzymatic activity and prevent DNA degradation. Samples were collected every 12 h from dry pitfall traps and pseudostem traps, which were distributed at 4-m intervals over the plots, and by direct capture. As indicated by King *et al.* [20], predation events occurring in traps remain a substantial concern for diet analyses. To reduce this possible source of error, we focused on direct capture, frequently collected predators in the traps, and excluded samples from traps that contained fragments of herbivores or other predators. Samples were placed in separate tubes in 96% ethanol; the tubes were temporarily kept in a portable cooler until they were transported to the laboratory and stored at −20°C.

### Feeding Trials and Positive Controls

To determine whether it was possible to detect *C. sordidus* DNA in the gut contents of predators, we collected 10 additional samples of *O. baurii* in the field by direct capture and placed them individually in tubes with moistened cotton and without food for 96 h. At this stage, seven samples were killed and then placed individually in clean tubes containing 96% ethanol and stored at −20°C; among them, three samples were analysed alone, and four samples were analysed after the addition of one *C. sordidus* egg before DNA extraction. The remaining three samples, which had been kept alive, were placed in new tubes with moistened cotton and one *C. sordidus* egg (one predator and one egg per tube); after 12 h, each individual of *O. baurii* had fed on the provided egg and was placed in a clean tube in 96% ethanol and stored at −20°C. In a “positive control” experiment, we also analysed the quantity of sequences recovered as a function of the number of *C. sordidus* eggs in a sample without predators; this experiment used 1 egg (n = 9), two eggs (n = 3), and three eggs (n = 3).

### Construction of the Mini-COI Bank of Sequences by SANGER Sequencing for Taxa Assignment

Predation was studied by amplifying and sequencing the mitochondrial *cytochrome c oxidase I* (COI) gene, which is widely used for species-level identification of animals [26]. Legs of two to four frozen samples of each taxon collected in the banana plantations (taxa and trapping methods are listed in **Table S1**) were used for the construction of the COI bank of sequences. Total DNA was extracted from legs with the DNeasy Blood and Tissue kit (Qiagen, Germany) following the manufacturer’s protocol. The long fragment of COI was amplified with the universal primers LCOI490 and HCO2198 [27] in a 20-μl volume containing 0.5 U of HotStarTaq plus DNA polymerase (Qiagen), 3 mM MgCl_2_, 400 μM of each dNTP, 10 μM of each primer, and 8 μl of arthropod DNA extract. After an initial activation of the DNA polymerase for 5 min at 95°C, the amplification was performed with 5 cycles of 1 min at 95°C, 1 min at 45°C, and 1.5 min at 72°C; followed by 30 cycles of 1 min at 95°C, 1 min at 48°C, and 1.5 min at 72°C; and a final extension of 5 min at 72°C. Amplicons were sequenced using the Sanger method on both strands and for each sample with the ABI3730XL analyser (Applied Biosystems) by the Macrogen sequencing service (Seoul, South Korea). Sequences were assembled and aligned with Geneious Pro 5.5.3 (Biomatters, New Zealand) before the mini-COI barcode was extracted. We deposited 15 sequences of COI in GenBank, and these included the sequences of two species previously not recorded in GenBank (see **Table S1**). The final mini-COI bank of sequences included the 15 sequences obtained from samples of the banana plantations and 20 additional COI sequences obtained from GenBank after the BLAST of the raw 454 sequences (sequences recovered with GenBank in **Table S3**).

### 454 Pyrosequencing of Mini-COI

We used a shortened fragment of COI, the mini-COI fragment (127 bp), which was amplified with primers Uni-MinibarF1 and Uni-MinibarR1 designed by Meusnier *et al.* [28]. Total DNA was extracted from the dissected gut contents or from the whole body (when body size was <1 cm) of the ground-dwelling predators with the DNeasy Blood and Tissue kit following the manufacturer’s protocol. To enable deconvolution of pooled 454 sequencing runs such that individual sequences could be traced back to a particular sample, we tagged the 5′ end of the PCR primers with different combinations of seven nucleotides (the tags are listed in **Table S4**). A total of 30 different tags enabled us to process the 572 samples of ground-dwelling predators for diet analyses and the 59 samples for positive controls (taxa, trapping methods, and positive controls are listed in **Table S2**). Deconvolution of the pooled sequences was performed with an exact search of the tag sequences. These tags differed in at least three nucleotides, which reduced the risk of incorrect assignment of sequences to sample ID in case of a sequencing error.

Amplification of mini-COI was performed in a 20-μl volume containing 0.5 U of HotStarTaq plus DNA polymerase (Qiagen), 3 mM MgCl_2_, 400 μM of each dNTP, 10 μM of each primer, and 8 μl of arthropod DNA extract. After an initial activation of the DNA polymerase for 5 min at 95°C, the mini-COI was amplified with 5 cycles of 60 s at 95°C, 60 s at 46°C, and 30 s at 72°C; 35 cycles of 60 s at 95°C, 60 s at 53°C, and 30 s at 72°C; and a final extension of 5 min at 72°C. Although blocking probes are often used to reduce the sequencing of predator DNA [29], they also make the procedure more cumbersome, especially when numerous taxa are analysed. In the current study, the pyrosequencing generated enough sequences so that prey could be identified even with over-representation of predator DNA sequences (see Results). An analysis of the migration of the PCR products obtained with tagged mini-COI primers enabled us to sort PCR products as a function of their signal intensity (null, low, medium, strong). PCR products from gut contents of ground-dwelling predators were concentrated in an oven at 35°C for 12 h. Then, we standardized the DNA concentration in all samples before pooling them in an equimolar solution. Pooled PCR products were purified with the QIAquick Gel Extraction Kit (Qiagen, Germany) and sequenced using the 454 GS FLX Titanium platform (Roche, Basel, Switzerland) of Beckman Coulter Genomics (Danvers, MA, USA).

### Bioinformatics Processing of Raw Sequences and Taxonomic Assignment

Pyrosequencing outputs were analysed using |SE|S|AM|E| BARCODE, a software designed to process the large amount of DNA sequences generated by pyrosequencing [for more details on the bioinformatics pipeline used, see 30,31]. The software loads a table in which the sequence of each tag is recorded for each sample and the FASTA file obtained from the pyrosequencing step. Sequences of interest are recovered after the BLAST of each raw sequence to a consensus sequence of the marker (threshold E-value = e^−2^, which is low to recover a maximum of sequences). This marker consensus sequence (127 bp) was obtained after the alignment [MUSCLE algorithm, 32] of all sequences of taxa found in the banana plantations (**Table S1**).

Mini-COI barcodes were assigned to taxa from the bank of sequences using the BLAST+ (E-value = e^−20^) and FASTA algorithm (85% similarity threshold) available in |SE|S|AM|E| BARCODE. Final barcoding identification was performed by assigning sequences with a Nearest Neighbour algorithm. Decision rules applied during the processing of sequences lead to an assignment to a higher taxonomic rank when the percentages of similarity were strictly equal between two or more species. Unique sequences were removed so that a minimum of two sequences was used for the barcoding identification. Sequences with fewer than 120 nucleotides (without primers) were discarded.

### Comparison of the Diet of the Ground-dwelling Predators in BSP vs. CCP

Although the number of sequences found for each prey was determined for each predator sample, this quantitative information could not be processed directly as indicated by Pompanon *et al.* [22]. The number of sequences obtained for a given prey was converted into binary information (presence/absence), and the trophic links were quantified by the number of predator samples among the population that were positive for each prey taxon. It is important to note that sequences assigned to a taxonomic rank higher than order were discarded from the analyses. Finally, we calculated the differences of frequency of consumption for each identified prey taxon between BSP and CCP and determined whether the differences were statistically significant using a Fisher’s exact test implemented within the statistical program R [33].

## Results

### Control Experiments

We first assessed our ability to amplify and detect *C. sordidus* mini-COI from 1, 2, or 3 *C. sordidus* eggs in a sample that lacked predator tissue or predator DNA. We correctly assigned the samples to *C. sordidus* for 14 of the 15 samples (see first three rows in **Table 1**). Nevertheless, no significant correlation was obtained between the number of eggs analysed and the number of sequences. We also performed trials in which each of three *O. baurii* was either fed with one *C. sordidus* egg and then analysed or in which each of four *O. baurii* was mixed with one *C. sordidus* egg and then analysed; although the detection rate was <100%, *C. sordidus* was detected in both cases (see rows 4–6 in **Table 1**). In negative controls (pure water), DNA of *Myospila lauta* Stein was detected (two sequences) (see rows 7 and 8 in **Table 1**) and was consequently excluded from further analyses because we suspected a possible cross-contamination (a total of 36 sequences of *M. lauta* were detected in four samples of *C. sexguttatus*).

**Table 1:** Positive controls, feeding trials, and negative controls.

Sample	n	Barcoding identification	Rank	Frequency (%)	Number of sequences		
					Total	Mean	SE
1 egg of *C. sordidus*	9	*Cosmopolites sordidus*	Species	89	127	14	4
2 eggs of *C. sordidus*	3	*Cosmopolites sordidus*	Species	100	116	39	19
3 eggs of *C. sordidus*	3	*Cosmopolites sordidus*	Species	100	164	55	21
*O. baurii* kept without food for 96 h	3	*Cosmopolites sordidus*	Species	0	0	0	0
*O. baurii* fed with 1 egg of *C. sordidus*	3	*Cosmopolites sordidus*	Species	33	4	1	1
*O. baurii* kept without food for 96 h +1 egg of *C. sordidus*	4	*Cosmopolites sordidus*	Species	50	61	15	16
Water (negative control)	10	Neoptera	Subclass	10	2	0.2	0.2
		*Myospila lauta*	Species	10	2	0.2	0.2

Positive controls (rows 1–3 in the table) were designed to estimate the number of sequences recovered as a function of the quantity of *C. sordidus* material assayed, which was varied by processing different numbers of *C. sordidus* eggs. The feeding trial (rows 4–6 in the table) was designed to determine whether *C. sordidus* DNA could be detected in the gut contents of a predator (*O. baurii* in this case) that had consumed one *C. sordidus* egg (in related treatments, the predator had not consumed a *C. sordidus* egg or was processed with one egg that it had not consumed). Negative controls (rows 7 and 8 in the table) consisted of ultra-pure HPLC grade water.

### Pyrosequencing Outputs and Resolution of the Mini-COI Barcode

**Figure 1:**
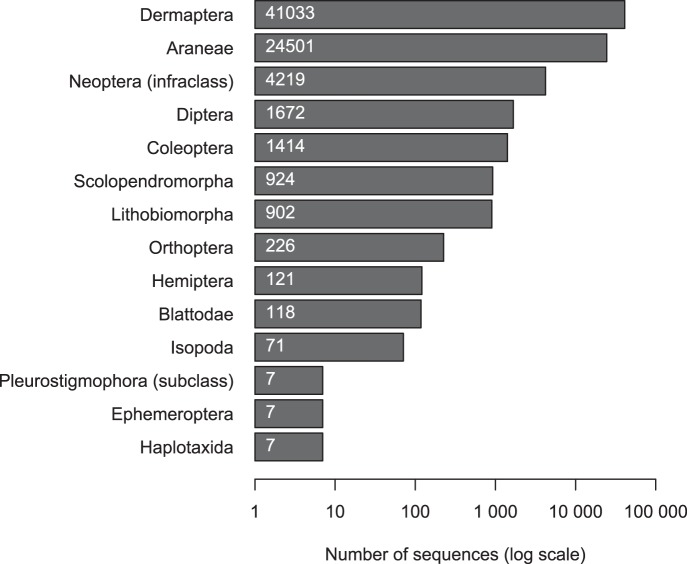
Number of sequences assigned to each order after mini-COI barcode sequencing. Data were derived from 454 pyrosequencing run, and sequences were assigned to taxa with SE|S|AM|E BARCODE (minimum of two sequences, BLAST+ E-value = e^−20^, FASTA 85% similarity threshold and Nearest Neighbour algorithm for the final identification). Bar charts indicate the total number of assigned sequences obtained for all taxa belonging to each taxonomic group. 42,915 sequences remained unassigned.

Among the 177,186 raw sequences obtained from the gut contents of predators, 118,180 sequences (67%) were identified as mini-COI marker, of which 75,313 sequences (43%) were successfully assigned to a taxon. A total of 56 distinct taxa belonging to 14 orders were identified (predator diet analyses and positive controls are indicated in **Figure 1**); 71.4% were identified to species, 80.3% to genus, and 91.0% to family. Among these taxa, nine were identified from sequences obtained in this study (**Table S2**) while the 47 remaining taxa were recovered from COI sequences recorded in GenBank (**Table S3**). Sequences of taxa belonging to the Lycosidae and to *E. caraibea* were very abundant (87% of the assigned sequences, **Table 2**). It is highly probable that these taxa were so efficiently amplified during the PCR step that they were later overrepresented in the samples. Consequently, we considered these taxa as a possible source of false positive prey detection, and we removed them from the diet analyses of predators.

**Table 2:** Frequency of taxa identified corresponding to each taxon analysed with the mini-COI barcodes.

Sample (*n*)	Barcoding identification	Rank	Frequency (*n*)	Number of sequences
Lycosidae (20)	*Trochosa*	Genus	100 (20)	18088
	*Pardosa*	Genus	65 (13)	2884
	*Pardosa milvina*	Species	15 (9)	9
	Lycosidae	Family	10 (2)	29
	*Pardosa amentata*	Species	5 (1)	11
	*Pardosa giebeli*	Species	5 (1)	2
	*Pardosa paludicola*	Species	5 (1)	3
	*Varacosa avara*	Species	5 (1)	4
*E. caraibea* (97)	*E. caraibea*	Species	100 (97)	39114
Scolopendra (6)	*Scolopendra*	Genus	100 (6)	544
	*Scolopendra mutilans*	Species	67 (4)	323
	Henicopidae	Family	50 (3)	864
	*Lamyctes hellyeri*	Species	50 (3)	8
	*Anopsobius giribeti*	Species	17 (1)	28
	*Scolopendra subspinipes*	Species	17 (1)	10
	*Scolopendra multidens*	Species	17 (1)	8
	Pleurostigmophora	Subclass	17 (1)	7
	*Otostigmus aculeautus*	Species	17 (1)	4
Cicadellidae (3)	Cicadellidae	Family	67 (2)	29
*C. sordidus* (15)	*Cosmopolites sordidus*	Species	93 (14)	407
Oniscidae (2)	Oniscidae	Family	50 (1)	69
Blattodae (2)	*Blatella germanica*	Species	100 (2)	101
*P. mellerborgi* (2)	*Polytus mellerborgi*	Species	100 (2)	365
*Gryllus* (1)	*Gryllus*	Genus	100 (1)	136
	*Orocharis saltator*	Species	100 (1)	59
	Gryllidae	Family	100 (1)	16
*A. castelnaui* (2)	*Tribolium castaneum*	Species	100 (2)	16
Lumbricidae (2)	(not assigned)	–	–	–
Rhinocricidae (2)	(not assigned)	–	–	–
Paradoxosomatidae (2)	(not assigned)	–	–	–
*O. baurii* (96)	(not assigned)	–	–	–
*C. sexguttatus* (103)	(not assigned)	–	–	–
*S. geminata* (155)	(not assigned)	–	–	–
*W. auropunctata* (108)	(not assigned)	–	–	–
Staphilinidae (10)	(not assigned)	–	–	–

Generated by 454 pyrosequencing, the table displays the taxonomic rank, the frequencies of samples and the corresponding sample size in brackets, and the number of sequences corresponding to taxa identified by barcoding and belonging to the same order of the taxa analysed. A frequency of 100% means that all samples of the taxa analysed had at least two sequences of the taxa identified by barcoding (BLAST+ with E-value = 10^−20^, FASTA with 85% similarity threshold, and Nearest Neighbour algorithm for the final identification).

### Identification of Prey from the Gut Contents of Ground-dwelling Predators

**Figure 2:**
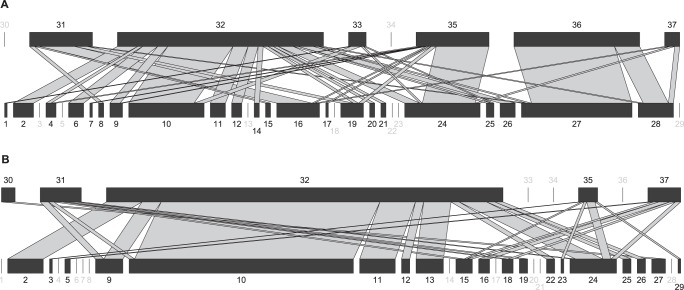
Bipartite food webs of predator-prey interactions on (A) bare soil, and (B) cover cropped banana plantation. For each web, lower bars represent relative abundance of consumed prey, and upper bars represent relative abundance of positive ground-dwelling predators, each drawn at different scale. The width of links between ground-dwelling predators and prey represents the frequency of consumption. Numbers in grey indicate unlinked taxa. Visualization was performed with the R package “bipartite” [50]. 1: *Anopheles claviger.* 2: *Anopheles nimbus.* 3: *Baetis rhodani.* 4: *Blatella germanica.* 5: *Calliphora vomitoria.* 6: *Carabidae* spp. 7: *Codophila varia*. 8: *Coridius chinensis*. *9*: *Cosmopolites sordidus*. 10: Diptera. 11: *Drosophila anceps*. 12: *Drosophila melanica*. 13: *Drosophila montana*. 14: *Gryllus*. 15: Hemiptera. 16: *Jalysus spinosus*. 17: *Nebria chinensis*. 18: *Neoneides muticus*. 19: *Nezara viridula*. 20: Oniscidae. 21: *Ophyra spinigera*. 22: *Periplaneta americana*. 23: *Podisus serieventris*. 24: *Polytus mellerborgi*. 25: *Resseliella yagoi*. 26: *Sarcophila*. 27: *Scolopendra*. 28: *Scolopendra mutilans*. 29: *Stephensioniella sterrei*. 30: Lycosidae. 31: *Camponotus sexguttatus*. 32: *Euborellia caraibea*. 33: *Odontomachus baurii*. 34: Scolopendridae. 35: *Solenopsis geminata*. 36: Staphilinidae. 37: *Wasmannia auropunctata.*

A total of 29 prey taxa were identified from the gut contents of the eight ground-dwelling predators taxa found in the banana plantations (**Figure 2**). DNA sequences of the banana weevil *C. sordidus* were recovered from samples of three species, with relatively low frequencies of consumption (frequency of consumption refers to the percentage of samples that were positive for DNA of the prey in question): the earwig *E. caraibea* (frequencies: BSP = 3%, CCP = 7%), the carpenter ant *C. sexguttatus* (CCP = 3%), and the fire ant *S. geminata* (BSP = 1%). The BLAST of sequences against the COI sequences recorded in GenBank enabled identification of dipteran arthropods that were not sampled in the plots during the study. We did not identify prey consumed by samples from the Scolopendridae family in either plot. Because of the absence of prey sequences in one of the two plots, the diet change could not be assessed for samples from the Lycosidae family, *O. baurii*, or the Staphilinidae family (frequencies of consumption are listed in **Table S5**).

### Difference in the Diets of Ground-dwelling Predators between BSP and CCP

**Figure 3:**
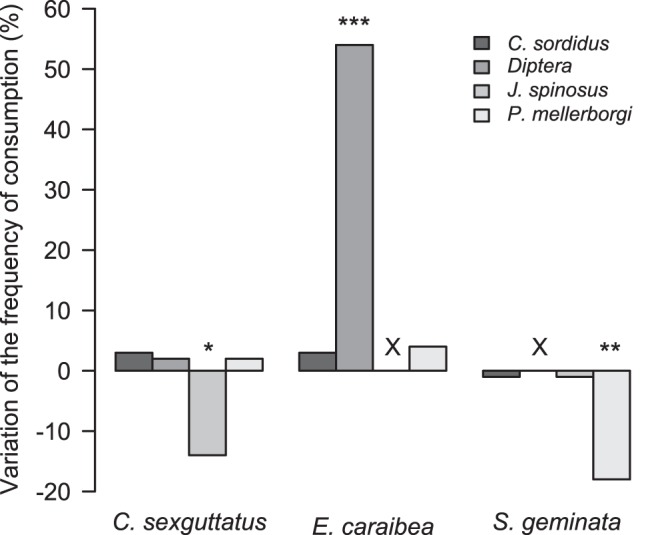
Diet changes of ground-dwelling predators between bare soil plot and cover cropped plot. The bar charts display the difference of frequencies of consumption for each prey calculated between the two plots, with the bare soil plot as a reference. The significance of the difference of frequencies of consumption observed between plots was assessed with a Fisher’s exact test. (***: *p*-value<0.0001; **: *p*-value<0.001; *: *p*-value<0.01). Black crosses above bar charts indicate that the prey was not detected in both treatments.

Twenty-nine prey taxa were identified from the gut contents of ground-dwelling predators. Among these, 22 prey taxa were identified in BSP while 19 were identified in CCP; 12 prey taxa were detected in both plots. Frequencies of prey detected from gut contents significantly differed in the two plots for some of the ground-dwelling predators (**Figure 3**). Interestingly, whereas *Jalysus spinosus* (Say) was detected as the main prey of the carpenter ant *C. sexguttatus* in BSP (14% positive), no sample of *C. sexguttatus* was positive for this prey in CCP (Fisher’s exact test, *p*-value = 0.0043). The frequency at which the earwig *E. caraibea* was positive for dipteran DNA was 26% in the BSP and 80% in the CCP (Fisher’s exact test, *p*-value <0.0001). The frequency at which the fire ant *S. geminata* was positive for DNA of the little banana weevil *Polytus mellerborgi* Heller was 21% in the BSP and 3% in the CCP (Fisher’s exact test, *p*-value = 0.0006). Samples of *C. sexguttatus* that were positive for banana weevil DNA were found only in CCP, while samples of *S. geminata* that were positive for banana weevil DNA were found only in BSP. *Cosmopolites sordidus* was detected in the gut contents from two samples of *E. caraibea* in BSP (*n* = 53) and from two other samples in CCP (*n* = 30).

## Discussion and Conclusions

We used a metabarcoding approach to analyse the gut contents of eight ground-dwelling predators in banana plantations. We demonstrated that the addition of a new primary resource in the agroecosystem modified the diet for some of the predators. This is one of few studies that has used metabarcoding to investigate the gut contents of the arthropod food web. The results suggest that this is a feasible approach for ecological studies and also revealed some issues that will require methodological adjustments.

The efficiency of the mini-COI PCR was highly variable depending on the predator taxa examined, and this remains a major problem in achieving comprehensive identification of the prey ingested by a predator. While some of the predator taxa (e.g., the Lycosidae) were over-represented in the amplicon set, others were not amplified at all (e.g., the Hymenoptera). Regarding the latter, Yu *et al.* [34] also recently reported difficulties with COI sequencing of hymenopteran samples with 454 technology. This problem indicates that optimization of PCR conditions and primers is essential. The combination of several barcodes [35] could be used to achieve a complete description of the predator diets. The differences in PCR efficiencies also resulted in our inability to include some taxa in the food web and confirmed that metabarcoding data cannot be interpreted in a quantitative manner [22]. Relating the number of DNA sequences detected to the amount of material ingested appears to be an intractable problem. The quantification of trophic interactions would be enhanced by coupling the metabarcoding approach with stable isotope analysis [36] that enables assessment of the quantity of material used for the construction of an organism [14]. A second problem was the large number of the sequences that were unassigned despite our effort to sample comprehensively and thus to include arthropod diversity from the studied sites in the bank of sequences. In addition, more than 4,000 sequences were assigned to the Neoptera infra-class level. Such uninformative identification may result from the incompleteness of the bank of sequences or from the incorrect identification of COI sequences recorded in GenBank [37]. The non-assignment of sequences should be prevented by an exhaustive and rigorous sampling in the studied ecosystem.

Despite the methodological shortcomings mentioned above (i.e., it must be borne in mind that the food webs described here are incomplete), metabarcoding is an excellent approach for inferring the food web from the natural environment and for detecting differences in its topology among treatments. These advantages were particularly appealing for our study, in which we aimed to detect differences in the predation of a major pest between two banana plantation management systems.

In the current study, the banana weevil *C. sordidus* was identified in the gut contents of three ground-dwelling predators: the earwig *E. caraibea,* the fire ant *S. geminata*, and the carpenter ant *C. sexguttatus*. These three species have previously been shown to feed on the banana weevil. In laboratory trials, earwigs of the genus *Euborellia* in Kenya [38] and other dermapterans in Indonesia [39] attacked *C. sordidus* eggs and larvae. The fire ant *S. geminata* is often described as an important generalist predator [40]–[42] and was observed to feed on *C. sordidus* in banana agroecosystems in Martinique [25]. Here, we showed that *S. geminata* workers that were directly sampled in the field were positive for *C. sordidus* DNA; however, the role of this species in the consumption of the pest may be underestimated in our study because ant workers usually carry prey to their nest to feed the colony [43]. Carpenter ants in the genus *Camponotus* were found in pseudostem leaf sheaths and leaf trash in Indonesia and were predicted to forage in the banana plant [39], which is the habitat of immature stages of *C. sordidus*. Here, the diet analysis of *C. sexguttatus* revealed the consumption of *C. sordidus* by ant workers that were trapped in the field. In contrast, although ants in the genus *Odontomachus* are thought to be predators of *C. sordidus* [44] and although *O. baurii* consumed *C. sordidus* eggs in a laboratory assay of the current study, we did not detect *C. sordidus* DNA in *O. baurii* trapped in the field. From these results, we identified three species of predators that could be considered for the control of *C. sordidus* populations in banana plantations. Two of the three species are ants, which are often assumed to play a key role in the regulation of *C. sordidus* in banana plantations [44]–[46].

In this study, we described the changes in the diets of generalist predators induced by plant diversification of a cropping system. We demonstrated that the use of a *B. decumbens* cover crop in banana plantations altered the arthropod food web, with significant changes in the frequency of consumption of some of the prey. Duyck *et al.* [47] found similar results based on stable isotope analyses, i.e., the trophic positions of generalist predators were changed by cover cropping. In the current study, the percentage of samples of the earwig *E. caraibea* that were positive for dipteran DNA was higher in CCP (80%) than in BSP (26%). This diet change suggests the *B. decumbens* cover crop probably increases the abundance of dipterans and thereby increased their consumption by *E. caraibea*. In sugarcane fields in Hawaii, weeds favour dipterans by providing food, shade, and resting areas [48]. Management of the *B. decumbens* cover crop by mowing is required to maintain the trade-off between the increase of predator densities and pest control. However, the increase in alternative prey (i.e., prey other than the target pest) in the diet of generalist predators exemplifies the processes that can dampen the positive effects of cover crops on pest regulation. In other words, the predators may increase consumption of non-pests without increasing consumption of pests.

In conclusion, it is essential to disentangle trophic interactions in order to achieve a better understanding of ecosystem resilience and persistence following disturbances [49], such as plant diversification [3]. DNA metabarcoding allows direct inference of trophic interactions and enables the assessment of arthropod diet. Although the method has limitations, including the inability to discriminate between direct predation, secondary predation, and scavenging [20], it has the potential to be very useful for describing arthropod food webs. Here, we identified new and unexpected trophic interactions in the predator–prey system in banana plantations. The accurate determination of trophic networks will challenge current models of trophic interactions and will contribute to food web theory and ecosystem management. In addition to its application to individual food webs, DNA metabarcoding could be used to link different food webs, such as those that describe micro-organisms, plants, arthropods, and larger animals.

## Supporting Information

Table S1 **List of taxa collected in banana plantations in order to sequence CO1 with SANGER method.** This sampling was performed to build the bank of sequence from individuals of the studying site. Sequences were aligned (MUSCLE algorithm) to build the consensus sequence used during the bioinformatics processing of raw sequences (Consensus sequence of mini-CO1∶5′-TTTATATTTTATTTTTGGARCTTGAGCAGGAATAGTAGGAACTTCATTAAGAATAHTTATTCGAGCAGAATTAGGAMAACCCGGATCATTAATTGGTGATGATCAAATTTATAATGTTATTGTTACA-3′).
(DOCX)

Table S2 **List of taxa collected in banana plantations for the 454 pyrosequencing.** Taxa were collected for diet analyses (ground dwelling predators, n = 572 samples), and for positive controls of the 454 pyrosequencing run (n = 59 samples). Positive controls are designed to check the efficiency of the pyrosequencing run.
(DOCX)

Table S3 **List of species identified with GenBank.** These taxa were recovered by blasting raw sequences derived from the 454 pyrosequencing run from the gut contents of ground-dwelling predators to GenBank database. Samples of these prey species were not collected during the sampling campaign designed in order to construct the bank of sequences. Identification to higher taxonomic rank results from an equal score calculated between two or more sequences of species recorded in GenBank, and the table displays taxa only for sequences identified to species rank.
(DOCX)

Table S4 List of tags used for forward and reverse primers. Each sample had a specific combination of tagged primers allowing an assignment of sequence to its respective sample ID. Each primer was added with a 7 nucleotide sequence (tag) at the 5′-end.
(DOCX)

Table S5 **Frequencies of consumption of prey by the ground-dwelling predators.** Resulting from the 454 pyrosequencing, the table displays the taxonomic rank, the frequencies of individuals and the corresponding number of individuals under brackets, and the number of sequences corresponding to prey identified by barcoding. The sample sizes for each predator and as a function of the treatment (bare soil and cover crop) are indicated under brackets. Barcoding identifications were validated with at least two sequences of the taxa identified (BLAST+ with e-value = 10^−20^, FASTA with 85% similarity threshold and Nearest Neighbour algorithm for the final identification).
(DOCX)
