# Supplementary material for: Cover Cropping Alters the Diet of Arthropods in a Banana Plantation: A Metabarcoding Approach
Source: PLoS One. 2014 Apr 2;9(4):e93740. doi: 10.1371/journal.pone.0093740 (PMC3973587; doi:10.1371/journal.pone.0093740)
Supplement: Table S1 — List of taxa collected in banana plantations in order to sequence CO1 with SANGER method. This sampling was performed to build the bank of sequence from individuals of the studying site. Sequences were aligned (MUSCLE algorithm) to build the consensus sequence used during the bioinformatics processing of raw sequences (Consensus sequence of mini-CO1∶5′-TTTATATTTTATTTTTGGARCTTGAGCAGGAATAGTAGGAACTTCATTAAGAATAHTTATTCGAGCAGAATTAGGAMAACCCGGATCATTAATTGGTGATGATCAAATTTATAATGTTATTGTTACA-3′). (DOCX) [file pone.0093740.s001.docx]

**Table S1. List of taxa collected in banana plantations in order to sequence CO1 with SANGER method**. This sampling was performed to build the bank of sequence from individuals of the studying site. Sequences were aligned (MUSCLE algorithm) to build the consensus sequence used during the bioinformatics processing of raw sequences (Consensus sequence of mini-CO1: 5’-TTTATATTTTATTTTTGGARCTTGAGCAGGAATAGTAGGAACTTCATTAAGAATAHTTATTCGAGCAGAATTAGGAMAACCCGGATCATTAATTGGTGATGATCAAATTTATAATGTTATTGTTACA-3’).

| **Taxon** | **Rank** | **n** | **Trapping method** | **Accession number** |
| --- | --- | --- | --- | --- |
| *Alegoria castelnaui* | Species | 4 | Pseudostem, direct capture | JX113274 |
| Blattodae | Order | 4 | Direct capture | JX113275 |
| *Camponotus sexguttatus* | Species | 4 | Direct capture | JX113276 |
| Cicadellidae | Family | 4 | Vacuum | JX113277 |
| *Cosmopolites sordidus* | Species | 3 | Pitfall, pseudostem | JX113278 |
| *Euborellia caraibea* | Species | 4 | Pseudostem, direct capture | JX113279 |
| *Gryllus* | Genus | 4 | Vacuum | JX113280 |
| Hydroporinae | Subfamily | 2 | Pseudostem | JX113281 |
| *Odontomachus baurii* | Species | 4 | Direct capture | JX113282 |
| Pentatomidae | Family | 2 | Vacuum | JX113283 |
| *Polytus mellerborgi* | Species | 4 | Pitfall, pseudostem | JX113284 |
| *Scolopendra* | Genus | 4 | Direct capture | JX113285 |
| *Solenopsis geminata* | Species | 4 | Pseudostem, direct capture | JX113286 |
| Staphilinidae | Family | 4 | Pseudostem, direct capture | JX113287 |
| *Wasmannia auropunctata* | Species | 4 | Pseudostem, direct capture | JX113288 |
