# Supplementary material for: Cover Cropping Alters the Diet of Arthropods in a Banana Plantation: A Metabarcoding Approach
Source: PLoS One. 2014 Apr 2;9(4):e93740. doi: 10.1371/journal.pone.0093740 (PMC3973587; doi:10.1371/journal.pone.0093740)
Supplement: Table S2 — List of taxa collected in banana plantations for the 454 pyrosequencing. Taxa were collected for diet analyses (ground dwelling predators, n = 572 samples), and for positive controls of the 454 pyrosequencing run (n = 59 samples). Positive controls are designed to check the efficiency of the pyrosequencing run. (DOCX) [file pone.0093740.s002.docx]

**Table S2. List of taxa collected in banana plantations for the 454 pyrosequencing.** Taxa were collected for diet analyses (ground dwelling predators, n=572 samples), and for positive controls of the 454 pyrosequencing run (n=59 samples). Positive controls are designed to check the efficiency of the pyrosequencing run.

| **Taxon** | **Rank** | **n** | **Trapping method** | **Analyses** |
| --- | --- | --- | --- | --- |
| *Camponotus sexguttatus* | Species | 103 | Direct capture | Diet |
| Cicadellidae | Family | 3 | Vacuum | Positive controls |
| *Cosmopolites sordidus* | Species | 31 | Laboratory rearing (egg) | Positive controls |
| *Euborellia caraibea* | Species | 83 | Pseudostem, direct capture | Diet |
| *Odontomachus baurii* | Species | 87 | Direct capture | Diet |
| *Odontomachus baurii* | Species | 10 | Direct capture | Positive controls |
| Gryllus | Genus | 1 | Vacuum | Positive controls |
| *Polytus mellerborgi* | Species | 2 | Pitfall, pseudostem | Positive controls |
| Blattodae | Order | 2 | Direct capture | Positive controls |
| Scolopendra | Genus | 6 | Direct capture | Diet |
| *Alegoria castelnaui* | Species | 2 | Pseudostem, direct capture | Positive controls |
| Lycosidae | Family | 20 | Direct capture | Diet |
| Lumbricidae | Family | 2 | Direct capture | Positive controls |
| Rhinocricidae | Family | 2 | Direct capture | Positive controls |
| Paradoxosomatidae | Family | 2 | Direct capture | Positive controls |
| Oniscidae | Family | 2 | Direct capture | Positive controls |
| Staphilinidae | Family | 10 | Pseudostem, direct capture | Diet |
| *Solenopsis geminata* | Species | 155 | Pseudostem, direct capture | Diet |
| *Wasmannia auropunctata* | Species | 108 | Pseudostem, direct capture | Diet |
