# Supplementary material for: Cover Cropping Alters the Diet of Arthropods in a Banana Plantation: A Metabarcoding Approach
Source: PLoS One. 2014 Apr 2;9(4):e93740. doi: 10.1371/journal.pone.0093740 (PMC3973587; doi:10.1371/journal.pone.0093740)
Supplement: Table S3 — List of species identified with GenBank. These taxa were recovered by blasting raw sequences derived from the 454 pyrosequencing run from the gut contents of ground-dwelling predators to GenBank database. Samples of these prey species were not collected during the sampling campaign designed in order to construct the bank of sequences. Identification to higher taxonomic rank results from an equal score calculated between two or more sequences of species recorded in GenBank, and the table displays taxa only for sequences identified to species rank. (DOCX) [file pone.0093740.s003.docx]

**Table S3. List of species identified with GenBank.** These taxa were recovered by blasting raw sequences derived from the 454 pyrosequencing run from the gut contents of ground-dwelling predators to GenBank database. Samples of these prey species were not collected during the sampling campaign designed in order to construct the bank of sequences. Identification to higher taxonomic rank results from an equal score calculated between two or more sequences of species recorded in GenBank, and the table displays taxa only for sequences identified to species rank.

| **Species** | **Accession number** |
| --- | --- |
| *Anopheles claviger* | HE614028 |
| *Anopheles nimbus* | HM022409 |
| *Baetis rhodani* | JN299155 |
| *Blatella germanica* | KC407709 |
| *Calliphora vomitoria* | KC617811 |
| *Codophila varia* | JN871581 |
| *Coridius chinensis* | JQ739179 |
| *Drosophila anceps* | DQ471598 |
| *Drosophila melanica* | EU390737 |
| *Drosophila montana* | DQ426756 |
| *Jalysus spinosus* | AY252906 |
| *Nebria chinensis* | HM180714 |
| *Neoneides muticus* | HQ105989 |
| *Nezara viridula* | CQ306225 |
| *Ophyra spinigera* | KC855280 |
| *Periplanata americana* | KC617846 |
| *Podisus serieventris* | HQ106268 |
| *Resseliella yagoi* | AB506014 |
| *Scolopendra mutilans* | AB672737 |
| *Stephensoniella sterreri* | GU902111 |
