# Supplementary material for: Cover Cropping Alters the Diet of Arthropods in a Banana Plantation: A Metabarcoding Approach
Source: PLoS One. 2014 Apr 2;9(4):e93740. doi: 10.1371/journal.pone.0093740 (PMC3973587; doi:10.1371/journal.pone.0093740)
Supplement: Table S4 — List of tags used for forward and reverse primers. Each sample had a specific combination of tagged primers allowing an assignment of sequence to its respective sample ID. Each primer was added with a 7 nucleotide sequence (tag) at the 5′-end. (DOCX) [file pone.0093740.s004.docx]

**Table S4.** List of tags used for forward and reverse primers. Each sample had a specific combination of tagged primers allowing an assignment of sequence to its respective sample ID. Each primer was added with a 7 nucleotide sequence (tag) at the 5’-end.

| **Tag name** | **Tag sequence** |
| --- | --- |
| 101 | ATCACTA |
| 102 | CTACTCG |
| 103 | CTACGTC |
| 104 | CTAGTAT |
| 105 | CTCATGC |
| 106 | CTCAGCT |
| 107 | CTGACGT |
| 108 | CTGTATG |
| 109 | ATCTAGT |
| 110 | CGAGTGA |
| 111 | CGTACAT |
| 112 | CGTCTAG |
| 113 | CGTCGCT |
| 114 | CGTGCTA |
| 115 | ATCTGAC |
| 116 | ATCGACG |
| 117 | ATGATCG |
| 118 | ATGTCGA |
| 119 | ATGCATC |
| 120 | ATGCTGT |
| 121 | ATGCGAG |
| 122 | ACAGTAG |
| 123 | ACAGCGT |
| 124 | ACTAGCA |
| 125 | ACTCAGT |
| 126 | ACTCGTC |
| 127 | ACTGATA |
| 128 | ACGTACA |
| 129 | ACGTCAG |
| 130 | AGACGTA |
