# Supplementary material for: Cover Cropping Alters the Diet of Arthropods in a Banana Plantation: A Metabarcoding Approach
Source: PLoS One. 2014 Apr 2;9(4):e93740. doi: 10.1371/journal.pone.0093740 (PMC3973587; doi:10.1371/journal.pone.0093740)
Supplement: Table S5 — Frequencies of consumption of prey by the ground-dwelling predators. Resulting from the 454 pyrosequencing, the table displays the taxonomic rank, the frequencies of individuals and the corresponding number of individuals under brackets, and the number of sequences corresponding to prey identified by barcoding. The sample sizes for each predator and as a function of the treatment (bare soil and cover crop) are indicated under brackets. Barcoding identifications were validated with at least two sequences of the taxa identified (BLAST+ with e-value = 10−20, FASTA with 85% similarity threshold and Nearest Neighbour algorithm for the final identification). (DOCX) [file pone.0093740.s005.docx]

**Table S5. Frequencies of consumption of prey by the ground-dwelling predators.** Resulting from the 454 pyrosequencing, the table displays the taxonomic rank, the frequencies of individuals and the corresponding number of individuals under brackets, and the number of sequences corresponding to prey identified by barcoding. The sample sizes for each predator and as a function of the treatment (bare soil and cover crop) are indicated under brackets. Barcoding identifications were validated with at least two sequences of the taxa identified (BLAST+ with e-value=10^-20^, FASTA with 85% similarity threshold and Nearest Neighbour algorithm for the final identification).

| **Predator sample** | **Treatment (*n*)** | **Prey** | **Rank** | **Frequency (*n*)** | **Number of sequences** |
| --- | --- | --- | --- | --- | --- |
| Lycosidae | Cover crop (19) | *Scolopendra* | Genus | 5 (1) | 2 |
|  | Bare soil (1) | NA | NA | NA | NA |
| *C. sexguttatus* | Cover crop (60) | Hemiptera | Order | 3 (2) | 7 |
|  |  | *Nezara viridula* | Species | 3 (2) | 7 |
|  |  | *Cosmopolites sordidus* | Species | 3 (2) | 6 |
|  |  | Diptera | Order | 2 (1) | 5 |
|  |  | *Polytus mellerborgi* | Species | 2 (1) | 2 |
|  |  | *Neoneides muticus* | Species | 2 (1) | 2 |
|  | Bare soil (43) | *Jalysus spinosus* | Species | 14 (6) | 21 |
|  |  | *Nezara viridula* | Species | 5 (2) | 15 |
|  |  | *Coridius chinensis* | Species | 2 (1) | 21 |
|  |  | *Scolopendra* | Genus | 2 (1) | 6 |
|  |  | Hemiptera | Order | 2 (1) | 3 |
| *E. caraibea* | Cover crop (30) | Diptera | Order | 80 (24) | 733 |
|  |  | *Anopheles nimbus* | Species | 13 (4) | 15 |
|  |  | *Drosophila anceps* | Species | 13 (4) | 22 |
|  |  | *Drosophila montana* | Species | 10 (3) | 14 |
|  |  | *Polytus mellerborgi* | Species | 10 (3) | 7 |
|  |  | *Cosmopolites sordidus* | Species | 7 (2) | 6 |
|  |  | *Drosophila melanica* | Species | 3 (1) | 9 |
|  |  | *Sarcophila* | Genus | 3 (1) | 4 |
|  |  | *Resseliella yagoi* | Species | 3 (1) | 2 |
|  |  | *Periplaneta americana* | Species | 3 (1) | 4 |
|  | Bare soil (53) | Diptera | Order | 26 (3) | 600 |
|  |  | *Anopheles nimbus* | Species | 8 (4) | 22 |
|  |  | *Polytus mellerborgi* | Species | 6 (3) | 12 |
|  |  | *Drosophila anceps* | Species | 6 (3) | 12 |
|  |  | *Sarcophila* | Genus | 6 (3) | 11 |
|  |  | *Calliphora vomitoria* | Species | 6 (3) | 6 |
|  |  | *Drosophila montana* | Species | 4 (2) | 12 |
|  |  | *Cosmopolites sordidus* | Species | 4 (2) | 7 |
|  |  | *Nezara viridula* | Species | 2 (1) | 9 |
|  |  | *Gryllus* | Genus | 2 (1) | 7 |
|  |  | *Ophyra spinigera* | Species | 2 (1) | 5 |
|  |  | *Scolopendra mutilans* | Species | 2 (1) | 4 |
|  |  | Oniscidae | Family | 2 (1) | 2 |
|  |  | *Resseliella yagoi* | Species | 2 (1) | 2 |
|  |  | Scolopendra | Genus | 2 (1) | 2 |
| *O. baurii* | Cover crop (11) | NA | NA | NA | NA |
|  | Bare soil (76) | Diptera | Order | 4 (3) | 15 |
|  |  | *Polytus mellerborgi* | Species | 1 (1) | 10 |
|  |  | *Anopheles claviger* | Species | 1 (1) | 4 |
|  |  | *Resseliella yagoi* | Species | 1 (1) | 3 |
| Scolopendridae | Cover crop (2) | NA | NA | NA | NA |
|  | Bare soil (4) | NA | NA | NA | NA |
| *S. geminata* | Cover crop (69) | *Polytus mellerborgi* | Species | 3 (2) | 7 |
|  |  | *Baetis rhodani* | Species | 1 (1) | 7 |
|  |  | *Stephensoniella sterreri* | Species | 1 (1) | 7 |
|  |  | *Nezara viridula* | Species | 1 (1) | 5 |
|  |  | *Podisus serieventris* | Species | 1 (1) | 3 |
|  | Bare soil (86) | *Polytus mellerborgi* | Species | 21 (18) | 201 |
|  |  | *Blattella germanica* | Species | 2 (2) | 8 |
|  |  | *Nezara viridula* | Species | 2 (2) | 5 |
|  |  | *Jalysus spinosus* | Species | 1 (1) | 10 |
|  |  | *Nebria chinensis* | Species | 1 (1) | 9 |
|  |  | Carabidae | Family | 1 (1) | 2 |
|  |  | *Cosmopolites sordidus* | Species | 1 (1) | 2 |
| Staphilinidae | Cover crop (0) | NA | NA | NA | NA |
|  | Bare soil (10) | *Scolopendra* | Genus | 40 (4) | 15 |
|  |  | *Scolopendra mutilans* | Species | 10 (1) | 4 |
| *W. auropunctata* | Cover crop (57) | *Jalysus spinosus* | Species | 4 (2) | 55 |
|  |  | *Neoneides muticus* | Species | 2 (1) | 7 |
|  |  | Hemiptera | Order | 2 (1) | 4 |
|  |  | *Polytus mellerborgi* | Species | 2 (1) | 4 |
|  |  | *Codophila varia* | Species | 2 (1) | 2 |
|  | Bare soil (51) | *Polytus mellerborgi* | Species | 2 (1) | 5 |
|  |  | *Scolopendra mutilans* | Species | 2 (1) | 2 |
|  |  | *Nezara viridula* | Species | 2 (1) | 2 |
